# Exploring Cultural Differences in the Recognition of the Self-Conscious Emotions

**DOI:** 10.1371/journal.pone.0136411

**Published:** 2015-08-26

**Authors:** Joanne M. Chung, Richard W. Robins

**Affiliations:** 1 Department of Psychology, University of California Davis, Davis, CA, United States of America; 2 Department of Developmental Psychology, Tilburg University, Tilburg, The Netherlands; University of Portsmouth, UNITED KINGDOM

## Abstract

Recent research suggests that the self-conscious emotions of embarrassment, shame, and pride have distinct, nonverbal expressions that can be recognized in the United States at above-chance levels. However, few studies have examined the recognition of these emotions in other cultures, and little research has been conducted in Asia. Consequently the cross-cultural generalizability of self-conscious emotions has not been firmly established. Additionally, there is no research that examines cultural variability in the recognition of the self-conscious emotions. Cultural values and exposure to Western culture have been identified as contributors to variability in recognition rates for the basic emotions; we sought to examine this for the self-conscious emotions using the University of California, Davis Set of Emotion Expressions (UCDSEE). The present research examined recognition of the self-conscious emotion expressions in South Korean college students and found that recognition rates were very high for pride, low but above chance for shame, and near zero for embarrassment. To examine what might be underlying the recognition rates we found in South Korea, recognition of self-conscious emotions and several cultural values were examined in a U.S. college student sample of European Americans, Asian Americans, and Asian-born individuals. Emotion recognition rates were generally similar between the European Americans and Asian Americans, and higher than emotion recognition rates for Asian-born individuals. These differences were not explained by cultural values in an interpretable manner, suggesting that exposure to Western culture is a more important mediator than values.

## Introduction

Darwin argued that emotions evolved to communicate an individual’s needs to conspecifics (“Help me!”), suggesting that every emotion should have an expressive signal reflecting its evolutionary origins. Using this provocative claim as a springboard, researchers have argued that all emotions necessarily have discrete, universally recognized nonverbal expressions (e.g., [1]). Until recently, this criterion appeared to be met by only the six basic emotions of anger, disgust, fear, joy, sadness, and surprise [2][3]. Other emotions, including the so-called “self-conscious” emotions [4], were often dismissed as mere variants, or combinations, of basic emotions (e.g., shame is a form of sadness).

Self-conscious emotions such as embarrassment, guilt, pride, and shame are unique because they require self-reflection and self-evaluation [5]. Thus, the self-conscious emerge later in development than the basic emotions do [6][7][8]. They are also cognitively complex [9][10], involving a primary appraisal (e.g., “is the event relevant to me?”), and a secondary appraisal (e.g., “how does this event make me feel about who I am?”). The self-conscious emotions are central for attaining goals that are social in nature [11][12]. The intersection between culture and the self-conscious emotions has been a fruitful area of research because of the role that culture is theorized to play in influencing self-concept (e.g., [13]), and in turn, various aspects of the self-conscious emotions. This research shows that there is cultural variation in social norms regarding the extent to which self-conscious emotions are valued and expressed [14][12], the ways in which they are used in socialization practices [15][16], and even how they are experienced [17][18][19].

Over the past decade and a half, a growing body of evidence suggests that embarrassment, shame, and pride might merit inclusion in the pantheon of emotions that have distinct, recognizable nonverbal expressions. Unlike the basic emotions, these self-conscious emotions can only be recognized when specific body postures and head movements accompany the facial features of each expression [20][21][22]. Specifically, embarrassment is associated with a downward gaze, lip press, non-Duchenne smile, head oriented to the side, and, at times, a face touch [20]; shame, with downward eye gaze, the head titled down, and, at times, a slumped body posture [22]; and pride, with a low intensity, non-Duchenne smile, an expanded posture with the head tilted back, and the arms raised above the head or akimbo [21].

Evidence that these expressions can be recognized in the United States at above-chance levels is fairly robust [e.g., [22]), at least in stimuli featuring Western targets. The studies that have examined the recognition of the self-conscious emotions in countries other than the United States suggests that embarrassment can be recognized in India [23]; shame in Burkina Faso [24], India and Japan [23][25]; pride in Burkina Faso [24], Italy [24], and perhaps South Korea [26], although this research was focused on triumph, an emotion that the authors conceptualized as a specific type of pride. The recognition rates ranged substantially across cultures and were frequently quite low. For example, in India, Haidt and Keltner [23] found below-chance recognition (15%) for one commonly studied variant of the shame expression that includes a tongue bite and significant but quite low recognition for shame expressions with the head and gaze down (20%) and with the face covered (35%). Similarly, Izard [25] found low recognition rates in India for shame expressions with the head and eyes lowered and with the head lowered but the eyes looking forward, finding a mean recognition rate of 32%; in Japan, the recognition rates were 41% for these two shame expressions. Finally, in Burkina Faso, Tracy and Robins [24] found a shame recognition rate of 34%, albeit for preliterate individuals with little or no exposures to Western media. In addition to the low recognition rates obtained in the extant research (at least for shame), and the small range of cultures studied, there are no studies that have comprehensively studied the self-conscious emotions, and contrasted them to recognition rates for basic emotions. Furthermore, given that Asia is one of the largest and most populated regions in the world, further research regarding emotion recognition in Asian cultures is important.

In general, studies examining the relationship between culture and emotion recognition have found the highest recognition rates for Western samples, and the lowest recognition rates in preliterate, non-Western samples. What might help explain this variability? Researchers have theorized that it may be due to such factors as cultural values or exposure to the culture in which the expressions were identified. For example, endorsing certain cultural values might facilitate or interfere with accurate recognition of certain emotion expressions [27]. Additionally, exposure, or having some general knowledge about cultural norms regarding emotion expression as well as being familiar with emotion-relevant facial physiognomy of the poser’s ethnic group, may influence the accuracy of emotion recognition [28]. Therefore, examining how either cultural values or exposure might be associated with recognition of the self-conscious emotions is worthwhile.

The present research had several aims. First, we sought to examine emotion recognition of the self-conscious emotions that have evidence for nonverbal expressions (i.e., embarrassment, shame, and pride) in an Asian country, South Korea (Study 1). To examine emotion recognition, we used the University of California, Davis Set of Emotion Expressions (UCDSEE) [22], a stimulus set featuring African and Caucasian posers. By using expressions posed by targets that are culturally distinct from that of the individual making the judgment, we hoped to provide a more conservative test of cross-cultural replication by examining the extent to which an emotion expression can be recognized on a person outside of one's culture. Second, we sought to examine variability in the recognition of the self-conscious emotions across three different cultural groups that vary in their exposure to Western culture: individuals who were born in Asia (currently living in the U.S.), Asian Americans (born and raised in the U.S.), and European Americans (born and raised in the U.S.) (Study 2). Finally, we tested whether cultural values might help explain differences in recognition rates across these three samples (Study 2). The present research adds to the emotion recognition literature by examining recognition of the self-conscious emotions in Asia and then exploring factors that may underlie cultural differences in recognition of the self-conscious emotions.

## Study 1

In Study 1, we examined the extent that individuals who were born and raised and currently living in South Korea were able to recognize nonverbal expressions assumed to represent the self-conscious emotions of pride, shame, and embarrassment. We also examined recognition rates for the basic emotions, to contrast them with recognition rates found for the self-conscious emotions, as well as with recognition rates found for basic emotions in other samples.

Like other East Asian cultures, South Korea is characterized by high endorsement of the cultural values of collectivism, power distance, uncertainty avoidance, and long-term orientation [29]. Endorsement of these values tell us that, on average, South Koreans are interdependent and deeply committed to the group, that status differences are widely accepted and highly consequential in South Korea, that there are rigid social norms for how people should act and feel, and that South Koreans are oriented towards planning for the future [29]. These values are in contrast to those endorsed in the United States [29]. If cultural values influence decoding rules for specific emotion expressions, then the stark differences between the values endorsed in South Korea from those endorsed in the United States suggest that emotion recognition rates might be generally different in South Korea.

What might we expect in terms of the recognition of the self-conscious emotions? In the extant literature, researchers have found that nonverbal expressions of positive emotions are generally recognized better than those for negative emotions [28][21]. Insofar as pride is considered a positive emotion, it could be that pride might still be afforded high recognition in South Korea, and that it might be recognized at higher rates than shame and embarrassment. Yet, it could be that cultural norms regarding the display and valuation of these emotions might influence recognition rates. Previous research suggests that there are rigid norms against the display of pride in Asian cultures, because pride is seen as undesirable [12], and socially disengaging, or disruptive to the cohesion of the group. Furthermore, other research indicates that shame is valued, discussed more, and seen as socially engaging in Asian cultures relative to Western ones [30][31]. Regarding embarrassment, researchers have reasoned that it is often expressed in reaction to a relatively inconsequential social wrongdoing, and serves an affiliative, appeasement function [20]. Put this way, embarrassment could also be considered to be a socially engaging emotion [30], and one that might be experienced more within South Korean culture, due to its hierarchical and collectivistic nature. Taken together, the literature suggests that within a South Korean sample, we might expect pride to be recognized more than shame and embarrassment. Additionally, while we expect that recognition rates for pride will be lower than those previously documented in research focused on Western cultures, we might expect that recognition rates for shame and embarrassment to be higher.

### Method

#### Participants

Eighty university students (22% women) ranging in age from 21 to 33 years (*Mdn* = 25 years) were recruited at Korea University in South Korea. Each participant was paid 5000 *won*, equivalent to $4.50 *USD*.

#### Stimuli

To assess emotion recognition, we used the University of California, Davis Set of Emotion Expressions (UCDSEE [22]), a stimulus set that includes FACS-verified versions of three self-conscious emotion expressions—pride, shame, and embarrassment—as well as the basic emotions of anger, disgust, fear, happiness, sadness and surprise. The UCDSEE includes 4 expressions of anger, disgust, fear, happiness, sadness, shame, and surprise; 7 expressions of embarrassment (4 with no face touch, 3 with a face touch); and 8 expressions of pride (4 with arms akimbo, 4 with arms raised). The UCDSEE features 4 expressors: an African male, an African female, a Caucasian male and a Caucasian female. For each photo, participants were asked to “decide which emotion, if any, you think is being expressed by the person in the photo. Please CIRCLE the emotion that best matches the emotion expressed by the person in the photo.” The response options were: *anger*, *disgust*, *embarrassment*, *fear*, *happiness*, *pride*, *sadness*, *shame*, *surprise*, *no emotion*, and *other (please specify)*. The “no emotion” and “other” options allowed participants to respond in an open-ended manner, which addresses concerns about the forced-choice format [32][33].

#### Procedure

The task was given in pencil-and-paper format. Participants individually viewed the stimuli (5 x 7 color photographs) in a randomized order. Participants then completed the emotion judgments using a rating form. The rating form was translated into Korean by a team of professional translators from Korea University. The translations were confirmed using an iterative process of translating and back-translating until the team had reached consensus. As in many languages, pride has two translations in the Korean language, *jarangseureoum* (with a neutral or positive valence), and *jaman* (negatively valenced). Thus, the rating form included 12 response options, and both *jarangseureoum* and *jaman* were considered correct for expressions of pride. After participants completed the emotion judgment task, they answered several items assessing demographics.

Before the data were analyzed, three bilingual coders examined the open-ended responses to determine whether any could be categorized into the existing emotion response options. The open-ended responses were only categorized when the coders were able to consensually assign the open-ended words to existing categories, which typically occurred when the open-ended responses were direct synonyms of the emotion category. The classification of the open-ended responses increased the recognition rates by less than 4% for all emotions except embarrassment. For embarrassment expressions, classifying s*ujubeum*, which means bashful or shy, as a correct response increased the recognition rate by 6.8%.

### Results

#### Recognition of the basic emotions

**Fig 1:**
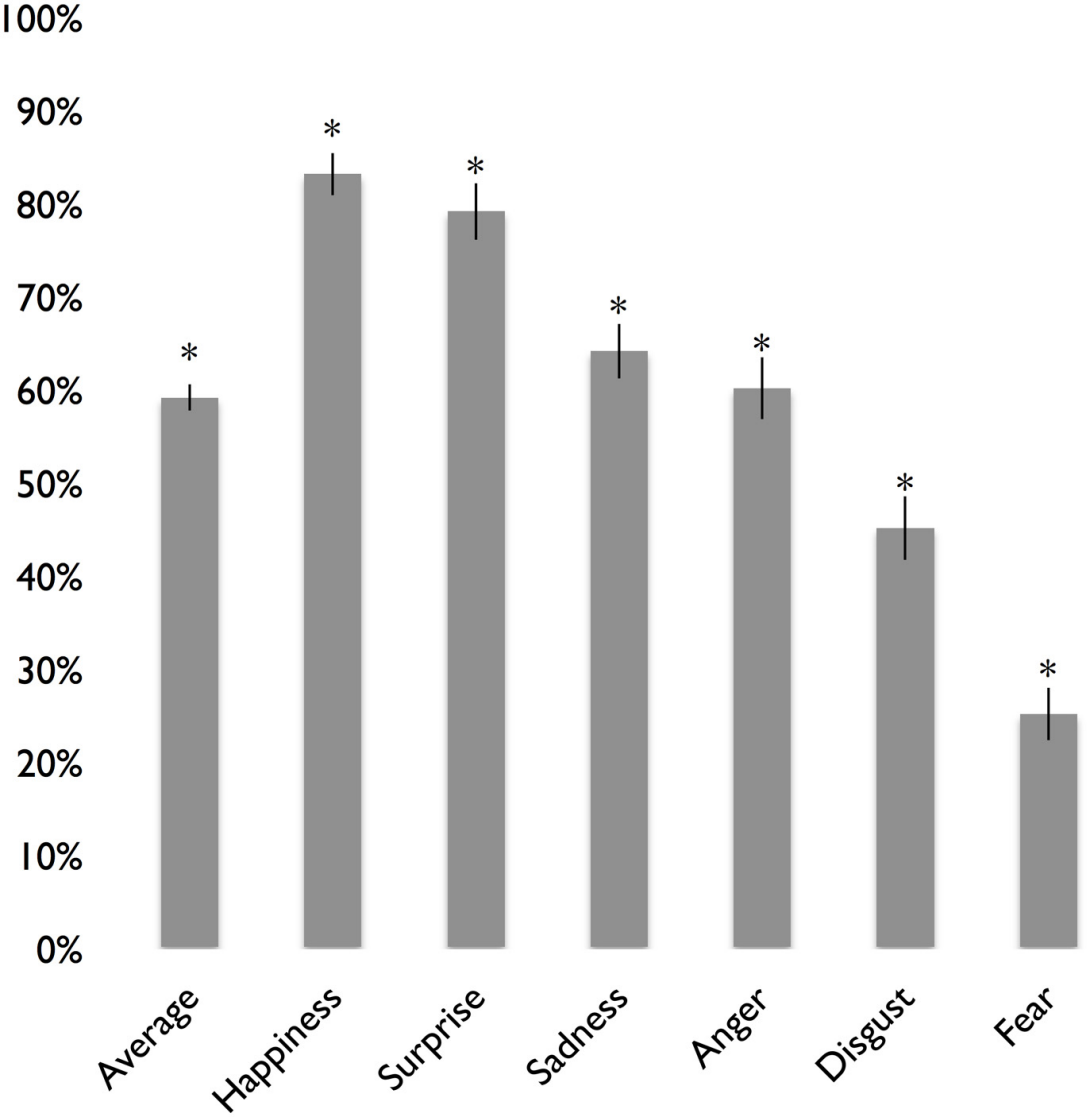
Mean Recognition Rates for the Basic Emotions in South Korea. *N* = 80. An asterisk (*) indicates recognition rates that are significant above chance. Standard error bars are shown.

Fig 1 shows mean recognition rates (averaged across posers) for emotion expressions of anger, disgust, fear, happiness, sadness, and surprise, as well as for the average across these expressions. The highest recognition rate was found for happiness, followed by surprise, sadness, anger, disgust and then fear. Based on binomial tests with chance set at 11%, the recognition rates for all of the basic emotions were significantly greater than chance (*p*s < .05), indicating that the nonverbal expressions for the basic emotions were cross-culturally recognized in our South Korean sample.

To better understand which emotion labels were being confused with emotion expressions and vice versa, we computed false alarm rates for each emotion expression. As Table 1 shows, happiness and sadness were frequently confused for pride and shame, respectively, anger and disgust were frequently confused with each other, and fear was often confused with several different emotions.

**Table 1:** Mean False Alarm Rates (Averaged Across Posers) for Basic Emotions in South Korean Sample.

Targeted expression and emotion label	Mean rate
Basic Emotions	
Happiness	
Happiness	**83%**
Pride	14%
Surprise	
Surprise	**79%**
Embarrassment	13%
Sadness	
Sadness	**64%**
Disgust	11%
Anger	7%
Shame	7%
Embarrassment	5%
Anger	
Anger	**60%**
Disgust	17%
Shame	7%
Sadness	5%
Fear	4%
Disgust	
Disgust	**45%**
Anger	34%
Embarrassment	5%
Pride	5%
Happiness	3%
Fear	
Fear	**26%**
Embarrassment	31%
Disgust	24%
Surprise	15%

*N* = 80. Values in boldface type represent hit (i.e., accuracy) rates, rather than false alarm rates. False alarm rates are shown up to 90 percent of responses.

#### Recognition of the self-conscious emotions

**Fig 2:**
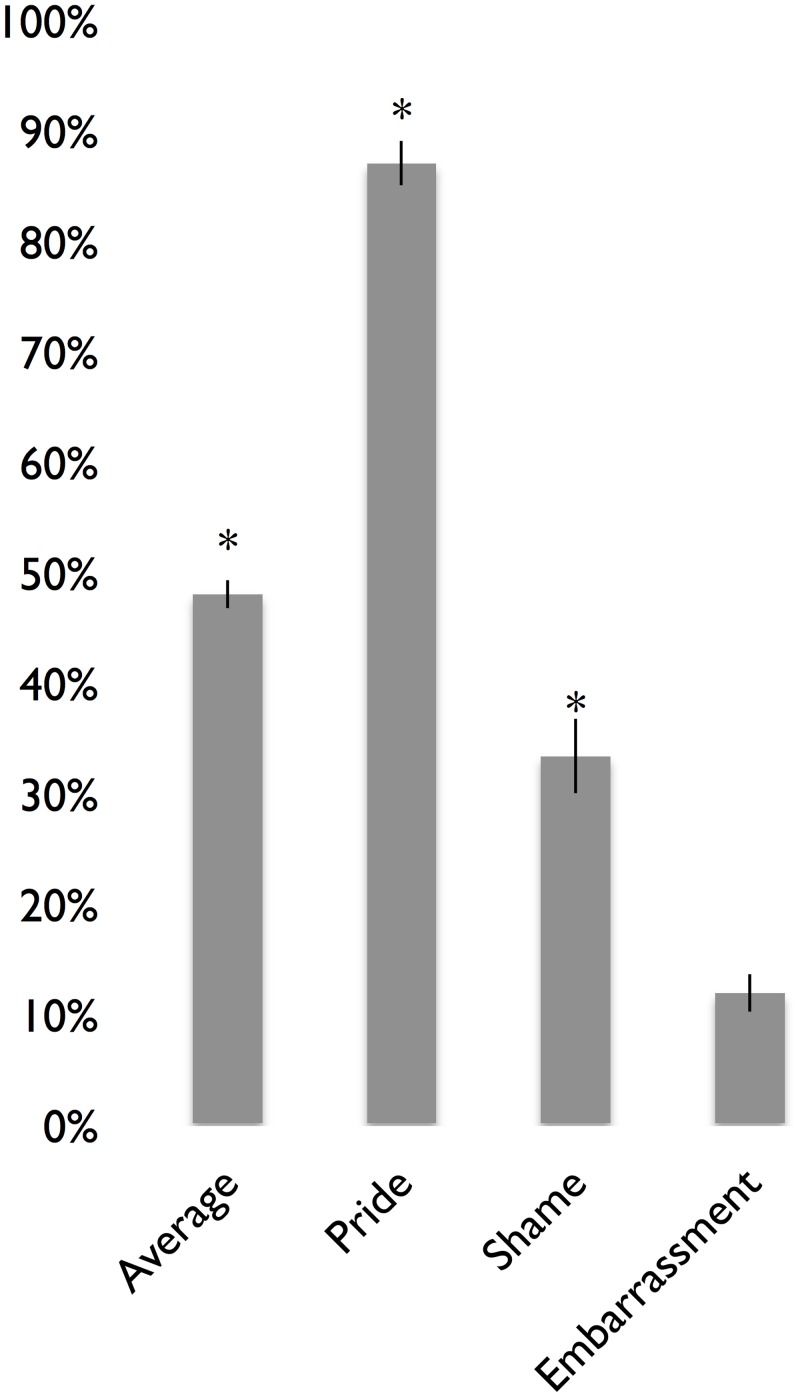
Mean Recognition Rates for the Self-Conscious Emotions in South Korea. *N* = 80. An asterisk (*) indicates recognition rates that are significant above chance. Standard error bars are shown.

Fig 2 shows mean recognition rates (averaged across posers) for emotion expressions of embarrassment, pride, and shame, as well as the average across these expressions. The highest recognition rate was found for pride, followed by shame, and then embarrassment. Based on binomial tests with chance set at 11% (based on the number of distinct emotion categories), the recognition rates for pride and shame were significantly greater than chance (*p*s < .05), but not for embarrassment, indicating that the nonverbal expressions of pride and shame were cross-culturally recognized in our South Korean sample, but that embarrassment was not. We also conducted binomial tests using the more stringent rate of 25%, a rate proposed by critics of forced-choice recognition studies who have argued that participants choose from four true emotion options defined by the two orthogonal dimensions of arousal and valence [33], suggesting a “true” chance guessing rate of 25%. Using this more stringent chance rate, the recognition rate for fear was no longer significantly greater than chance.

Recalling that there are two differently valenced words for pride in the Korean language, we examined the extent to which each response category was used for each pride photograph, shown in Table 2. In general, the neutral or positively valenced word for pride, *jarangseureoum*, was chosen more frequently as a response option than the negatively valenced word, *jaman*, with the exception of the expression posed by the Caucasian female with arms akimbo, indicating that when our Korean participants were looking at a pride expression, they were more likely to perceive it as a positive display of pride than a negative display of pride. The mean recognition rate for pride with arms akimbo (92%) differed significantly from the mean recognition rate for pride with arms raised (82%), *t*(79) = 3.71, and both recognition rates were significantly greater than chance, *p* < .05, suggesting that our Korean participants were more likely to judge nonverbal expressions of pride as such when the poser’s arms were akimbo. To examine whether emotion recognition rates of pride and embarrassment differed by photograph, we ran repeated measures ANOVAs, entering photograph as the independent variable, and emotion recognition rates as the dependent variable. For pride, the effect of photograph was significant, *F*(7, 553) = 7.72, *p* < .001, *η*_*p*_^*2*^ = .09, indicating that recognition rates varied by photograph (See Table 1 for hit rates for each photograph). We conducted post-hoc pairwise comparisons using a Bonferroni correction, and found that recognition of the expression posed by the African male, with arms raised, was significantly different from that of the other photographs, with the exception of the expression posed by the Caucasian female, with arms raised. For embarrassment, the effect of photograph was not significant, *F*(6, 474) = 1.72, *ns*, *η*_*p*_^*2*^ = .02, indicating that recognition rates did not significantly vary by photograph.

**Table 2:** South Korean Participants’ Use of Differently Valenced Korean Pride Words for Pride Photographs

	Pride Word		
	*jarangseureoum*	*jaman*	
	(Neutral/positive valence)	(Negative valence)	Averaged
Photo 29 Caucasian female, arms raised	80%	5%	85%
Photo 23 Caucasian male, arms raised	76%	16%	93%
Photo 10 African female, arms raised	78%	8%	86%
Photo 03 African male, arms raised	53%	14%	66%
Photo 04 Caucasian female, arms akimbo	28%	68%	95%
Photo 17 Caucasian male, arms akimbo	63%	26%	89%
Photo 34 African female, arms akimbo	68%	26%	94%
Photo 40 African male, arms akimbo	66%	23%	89%

*N* = 80.

The mean recognition rate for embarrassment expressions with a face touch (15%) did not differ significantly from the mean recognition rate for embarrassment expressions without a face touch (9%), *t*(79) = -1.92, *ns*, and neither recognition rate was significantly greater than chance, *p >* .05, indicating that regardless of the posturing of the body, expressions of embarrassment were not recognized well.

To better understand which emotion labels were being confused with emotion expressions and vice versa, we computed false alarm rates for each emotion expression. As Table 3 shows, pride and shame were frequently confused for happiness and sadness, respectively, and embarrassment was frequently confused with several different emotions.

**Table 3:** Mean False Alarm Rates (Averaged Across Posers) for Self-Conscious Emotions in South Korean Sample.

Targeted expression and emotion label	Mean rate
Pride	
Pride	**87%**
Happiness	10%
Shame	
Shame	**33%**
Sadness	50%
No Emotion	9%
Embarrassment	
Embarrassment	**12%**
Happiness	24%
Shame	19%
Other	15%
Pride	16%
Sadness	7%

*N* = 80. Values in boldface type represent hit (i.e., accuracy) rates, rather than false alarm rates. False alarm rates are shown up to 90 percent of responses.

#### Examining differences in recognition rates

Recognition rates across the basic emotions (*M* = 82%) were significantly higher than recognition rates across the the self-conscious emotions (*M* = 70%), *t*(482) = 15.13, *p* < .05. Overall recognition rates, averaged across all emotions, did not differ significantly for male (*M* = 51%) versus female (*M* = 50%) participants, *t*(63) = -.32, *ns*. In addition, expressions posed by Caucasian American targets (*M* = 55%) were recognized at similar rates as expressions posed by African targets (*M* = 54%), *t*(79) = -.77, *ns*. However, we did find a small gender effect for the poser; expressions posed by female targets (*M* = 56%) were better recognized than expressions posed by male targets (*M* = 52%), *t*(79) = 3.07, *p* < .05. Note, however, that this effects is quite small and could simply reflect the idiosyncrasies of the four posers used to create the UCDSEE stimuli, rather than generalizable differences in the degree to which expressions posed by men vs. women can be accurately recognized.

### Discussion

In Study 1, we examined recognition of the self-conscious emotions, as well as of the basic emotions, in South Korea. Several findings are noteworthy, and in some cases support the extant literature and in other cases raise questions about the prevailing view.

First, the findings provide additional evidence that the pride expression can be recognized cross-culturally. Despite the Asian emphasis on modesty and social norms against overt displays of pride [12], which likely lead to frequent regulation of the expression, we found that the pride recognition rate in South Korea was comparable to what has been previously found in the United States and Italy [24], and that it had the highest recognition rates, surpassing happiness, which is in contrast from what has been found in other cultures. Although Tracy and Robins’ [24] study of culturally isolated, preliterate individuals in Burkina Faso provides compelling evidence for the universality of the pride expression, showing that it is also recognized in Asia is important, especially because the emotion recognition process, as well as the self-evaluative processes that elicit pride, are assumed to be somewhat unique.

Second, the results suggest that the shame expression can be recognized at above-chance levels, but at quite low rates (33% in the present study), especially when contrasted to those found in the United States [20][22]. One interpretation of the overall pattern of findings, in both the present and previous studies, is that shame does not have an entirely distinct cross-culturally recognized expression. That is, although the presumed shame expression (and its assorted variants) clearly connotes something about shame (as evidenced by the above-chance recognition found in most studies), it may lack strong discriminant validity. Consistent with this interpretation, shame expressions were just as likely to be labeled sadness as shame in South Korea, India [23], and Burkina Faso [24]. An alternative explanation is that shame recognition is low in highly collectivistic cultures like South Korea, India, and Burkina Faso because the expression is so frequently regulated that it gets expressed in myriad, complex ways, making it difficult for members of these culture to discern the underlying prototypical expression. Further research is needed to tease apart these possibilities.

Third, in contrast to previous research in the United States [20][22], we failed to find significant recognition for the expression of embarrassment. However, the low recognition rate may reflect a cross-cultural difference in the emotion lexicon. Specifically, in both Korean and Oriya (the Indian language studied by Haidt and Keltner [23]), there is no single word for the emotional state of embarrassment, but rather a set of words that are loosely associated with embarrassment, shame, shyness, modesty, humility, and related concepts. Consequently, in these cultures, the mapping of the nonverbal expression onto the emotion label may not be as clear-cut as in the United States and other English-speaking cultures, where there is a single word that refers to a distinct emotional state. Thus, the low recognition rate for embarrassment may reflect the fact that the Korean translation of embarrassment encompasses a heterogeneous range of experiences, values, and tendencies that do not have clear nonverbal expressions (e.g., humility). Consistent with this interpretation, participants in the present study generated a wide range of open-ended responses to the embarrassment photos. We coded the response of *sujubeum* (shy/bashful) as correct, but another common response, *buggeureoum*, was not counted as correct because it can mean both embarrassment and shame (although it was exclusively used in response to embarrassment expressions). When we counted *buggeureoum* as a correct response, the recognition rate for embarrassment reached 25%, which is still quite low but above chance, *p* < .05.

Fourth, Study 1 provides support for the cross-cultural validity of the UCDSEE, but with some caveats. Recognition rates for the basic emotions were generally lower than have been previously found in the United States and higher than recognition rates found in Burkina Faso [24][22]. Interestingly, the recognition rates for fear and disgust were comparable to those previously found in Burkina Faso, which is surprising given that we would expect South Korean college students, with some exposure to Western media (and therefore to displays of the emotion expressions), to show higher recognition rates than the more culturally isolated Burkinabe. It is not clear whether the low recognition rates for these two emotions reflect limitations of the UCDSEE stimuli (i.e., that the Caucasian American and African actors were clearly of different cultural backgrounds than the participants) or a more general inability of South Korean participants to recognize fear and disgust in posed expressions developed in the United States. However, it is important to note that these findings are consistent with previous research in East Asian cultures; Matsumoto [27] found that Japanese participants were worse than their American counterparts at recognizing fear and disgust. Although the low recognition rates for fear and disgust held for both Caucasian and African targets, it would be interesting to examine whether using Asian targets would yield higher recognition rates as a result of an in-group advantage (e.g., [28]).

In summary, Study 1 contributes to the literature on the cross-cultural recognition of self-conscious emotions by examining them within South Korea, and finding evidence for cross-cultural recognition of pride and shame, but not embarrassment.

## Study 2

Because the primary aim of Study 1 was to examine recognition of nonverbal expressions for the self-conscious emotions within South Korea, we did not assess cultural variables. Yet, the findings from Study 1 raise the question of how culture might influence the recognition of self-conscious emotions. As a first step, we compare recognition rates in three groups of participants. Because we are particularly interested in Asian culture (given that Study 1 was conducted in South Korea), we focus on three cultural groups within the U.S.—Asian-born individuals currently living in the U.S. (i.e., individuals who had immigrated to the U.S., or were currently in the U.S. to attend college), Asian Americans who were born and raised in the U.S. (i.e., second generation and later individuals), and European Americans who were born and raised in the U.S. We expected the Asian-born group to have the lowest overall emotion recognition rates and the European American group to have the greatest overall emotion recognition rates. Additionally, because the primary distinction between the self-conscious emotions and the basic emotions is that they necessarily involve self-evaluation of one’s actions or characteristics in reference to socio-cultural norms [21], we expected recognition rates for self-conscious emotions to vary more than has been shown for the recognition rates for basic emotions, to the extent that self-evaluative processes vary according to cultural background [13] even within the U.S. [35].

Cultural values—the beliefs that people have about how to think, feel, and interact with others—have been discussed as a possible explanation for cultural variability in emotion recognition rates. Matsumoto [36] found that a Japanese sample had lower recognition rates than an American sample for the negative emotions of fear, anger, and disgust, suggested that this may be due to the cultural emphasis of the Japanese on collectivism and power distance, theorizing that this was due to unwillingness to perceive emotions that might be disruptive to group cohesion and rigid norms. Matsumoto [27] later found that variability in emotion recognition rates across several countries was associated with cultural dimensions. Specifically, he found that recognition of anger, disgust, and fear was negatively associated with the dimension of power distance, and recognition of anger, fear, and sadness was positively associated with the dimension of individualism [27]. This suggests that recognition of negative emotions varies with cultural values; the more hierarchical or collectivistic a culture is, the less the expressions of negative emotions might be recognized. To examine cultural values, we assessed the extent to which individuals endorsed cultural values that have been identified as being important to Asian cultures, such as collectivism, conformity to norms, emotional self-control, family recognition through achievement, humility, and loss of face [37][38]. We expected that our three cultural groups might differ in their degree of endorsement of Asian cultural values, with Asian-born individuals showing the highest endorsement of Asian values and European Americans showing the least. To the extent that the groups within our U.S. sample might differentially endorse Asian cultural values, we expected that those who were more endorsing of these values might also demonstrate lower recognition for the negative emotions than those were less endorsing of these values, and call this the Cultural Value Hypothesis. For the Cultural Values Hypothesis to be supported, all three groups should show significantly different recognition rates, with the Asian-born individuals endorsing these values the most, and the European Americans endorsing them the least, and these group differences should be explained by group differences in cultural values; that is, cultural values should entirely explain away the group differences. Regarding which values might contribute to group differences, we were most interested in examining how collectivism and conformity to norms might contribute to variability in emotion recognition rates, specifically for the negative emotions (i.e., the self-conscious emotions of embarrassment and shame, and the basic emotions of anger, disgust, fear, and sadness), based on previous research [36][27]. The contribution of the cultural values of emotional control, family recognition through achievement, humility, and loss of face were examined in an exploratory manner. Moreover, in line with our reasoning concerning the implications that culture has for the self (e.g., if culture greatly influences self-concept [13], then perhaps the self-conscious emotions are more culturally variable than the basic emotions are), we might expect that cultural values might be especially relevant in accounting for cultural variability in the recognition rates for the self-conscious emotions, and thus explain more variance, relative to recognition rates for the basic emotions.

In addition to cultural values, exposure to the culture in which a stimulus set is derived has been found to lead to higher emotion recognition rates. In a meta-analysis examining culture and emotion recognition variability, Elfenbein and Ambady [28] found that emotion recognition rates were higher when participants judged expressions posed by members of their own cultural group, or cultural groups with which they had considerable exposure to. For example, European and American participants showed higher recognition rates than Asian or African participants for stimuli featuring American expressors (e.g., [39] and [25]). Similarly, Ethiopian participants who were more exposed to Western culture had higher emotion recognition rates than those who were less so [40]. The UCDSEE was derived in North America and features targets that are representative of two common racial groups, Caucasian and African, in the West. Thus, we might expect that those more exposed to Western cultural norms and people (i.e., the European Americans and Asian Americans in our sample) would have higher emotion recognition rates than those who are less so (i.e., the Asian-born individuals in our sample), and refer to this as the Exposure Hypothesis. For the Exposure Hypothesis to be supported, then the Asian-born individuals in our sample should show lower recognition rates than the Asian American and European-American individuals, but the latter two groups should not differ from each other (because they were both born and raised in the United States and presumably do not differ in exposure to Western culture); moreover, variability in cultural values, either between- or within-groups, should not be linked to group or individual variability in emotion recognition.

Study 2 sought to examine groups that would show variability in their emotion recognition rates, exposure to Western culture, and endorsement of Asian cultural values. As in Study 1, we used the UCDSEE [22]. We tested a variety of cultural variables to examine what factors might help to explain cultural variability in emotion recognition rates. Therefore, Study 2 contributes to the emotion recognition literature by examining cultural variability in the recognition of self-conscious emotions within the United States, and by examining cultural factors that may underlie cultural differences in emotion recognition of the self-conscious emotions.

### Method

#### Participants

Four-hundred and eighty-two university students (65% women) ranging in age from 18 to 38 years (*Mdn* = 19 years) were recruited from the Psychology subject pool at the University of California, Davis, and included undergraduate students enrolled in both introductory and advanced courses. Each participant received 1 course credit corresponding to 1 hour of participation. Participants self-reported their ethnic background and where they were born and raised. For the present study, participants were categorized as European American (*N* = 195), Asian American (*N* = 191), and Asian-born (*N* = 96). Participants were categorized as being European American and Asian American if they self-reported being born and raised in the United States. European Americans self-reported their ethnicity as Caucasian, and Asian Americans self-reported their ethnicity as Asian. Asian-born participants reported being born and raised in an Asian country, and who reported their ethnicity as Asian (See Table 1 in the S1 Appendix for more detailed demographic information on this group). The three groups did not differ in the proportion of men and women, χ^2^(2, *N* = 481) = .03, *ns*.

#### Stimuli

As in Study 1, we used the UCDSEE [22], which includes 47 FACS-verified expressions of anger, disgust, embarrassment, fear, happiness, pride, sadness, shame, and surprise. The instructions were the same as in Study 1, except that we added “guilt” as a response option. See Table 4 for means and standard deviations.

**Table 4:** Descriptive Statistics for Emotion Recognition Rates in the U.S. Sample by Cultural Group.

	Asian-born		Asian American		European American	
	(*N* = 96)		*(N* = 191)		(*N* = 195)	
Variable	*M*	*SD*	*M*	*SD*	*M*	*SD*
Basic Emotions	.76^a^	.13	.81^b^	.11	.85^c^	.09
Happiness	.96^a^	.13	.99^b^	.08	.99^b^	.07
Surprise	.94^a^	.13	.94^a^	.13	.95^a^	.10
Sadness	.64^a^	.25	.70^b^	.23	.72^b^	.23
Anger	.82^a^	.25	.93^b^	.16	.95^b^	.13
Disgust	.70^a^	.27	.82^b^	.23	.88^c^	.18
Fear	.49^a^	.31	.48^a^	.33	.63^b^	.31
Self-Conscious Emotions	.59^a^	.18	.74^b^	.15	.73^b^	.15
Pride	.85^a^	.21	.91^b^	.14	.91^b^	.13
Shame	.26^a^	.31	.50^b^	.32	.53^b^	.32
Embarrassment	.46^a^	.34	.67^b^	.31	.62^b^	.31

*N* = 482. Subscripts denote significant group differences, *p* < .05.

#### Measures of Cultural Variables

Asian American Values Scale—Multidimensional (AAVS-M [37]). Asian cultural values refer to beliefs that are socially desirable and normative in Asian cultural contexts. Asian-born individuals endorse these values more strongly than Asian Americans who are several generations removed from immigration. The AAVS-M assesses five types of cultural values identified to be salient within Asian contexts, and consists of 42 items with a 7-point response scale ranging from 1 (*strongly disagree*) to 7 (*strongly agree*) that measure five value dimensions: collectivism (“The welfare of the group should be put before that of the individual.”), conformity to norms (“One should not do something that is outside of the norm.”), emotional self-control (“It is more important to behave appropriately than to act on what one is feeling.”), family recognition through achievement (“Failing academically brings shame to one’s family.”), and humility (“One should not sing one’s own praises.”).

Loss of Face Scale (LOFS [38]). Loss of face is the tendency to be concerned with preventing the loss of a positive impression others have of that individual. Loss of face has been identified as central to social interactions in Asian cultural contexts. The LOFS consists of 21 items with a 7-point response scale ranging from 1 (*strongly disagree*) to 7 (*strongly agree*). An example item is, “I carefully watch others’ actions before I do anything.”

See Table 5 for means, standard deviations, and coefficient alphas, and Table 2 in the Appendix for intercorrelations among the cultural variables.

**Table 5:** Descriptive Statistics for Cultural Variables in the U.S. Sample by Cultural Group.

	Asian-born (*N* = 96)			Asian American (*N* = 191)			European		
							American (*N* = 195)		
Variable	*M*	*SD*	*α*	*M*	*SD*	*α*	*M*	*SD*	*α*
Collectivism	4.22^a^	.78	.70	4.21^b^	.70	.69	4.04^b^	.89	.82
Conformity to Norms	4.31^a^	.76	.65	3.86^b^	.82	.73	3.60^c^	.98	.82
Emotional Self-Control	3.93^a^	.74	.61	3.61^b^	.76	.73	3.31^c^	.93	.83
Family Recognition Through Achievement	4.43^a^	.94	.90	4.56^a^	.75	.86	4.12^b^	.88	.89
Humility	3.94^a^	.73	.52	3.89^a^	.78	.70	3.89^a^	.92	.83
Loss of Face	4.34^a^	.91	.91	4.56^b^	.76	.86	4.34^a^	.84	.88

#### Procedure

The task was given in online questionnaire format. Participants individually viewed the UCDSEE stimuli and completed the emotion judgments in online questionnaire format. Participants then completed the AAVS-M, and LOFS. Participants then answered several items assessing demographics.

### Results

#### Recognition of the basic emotions

**Fig 3:**
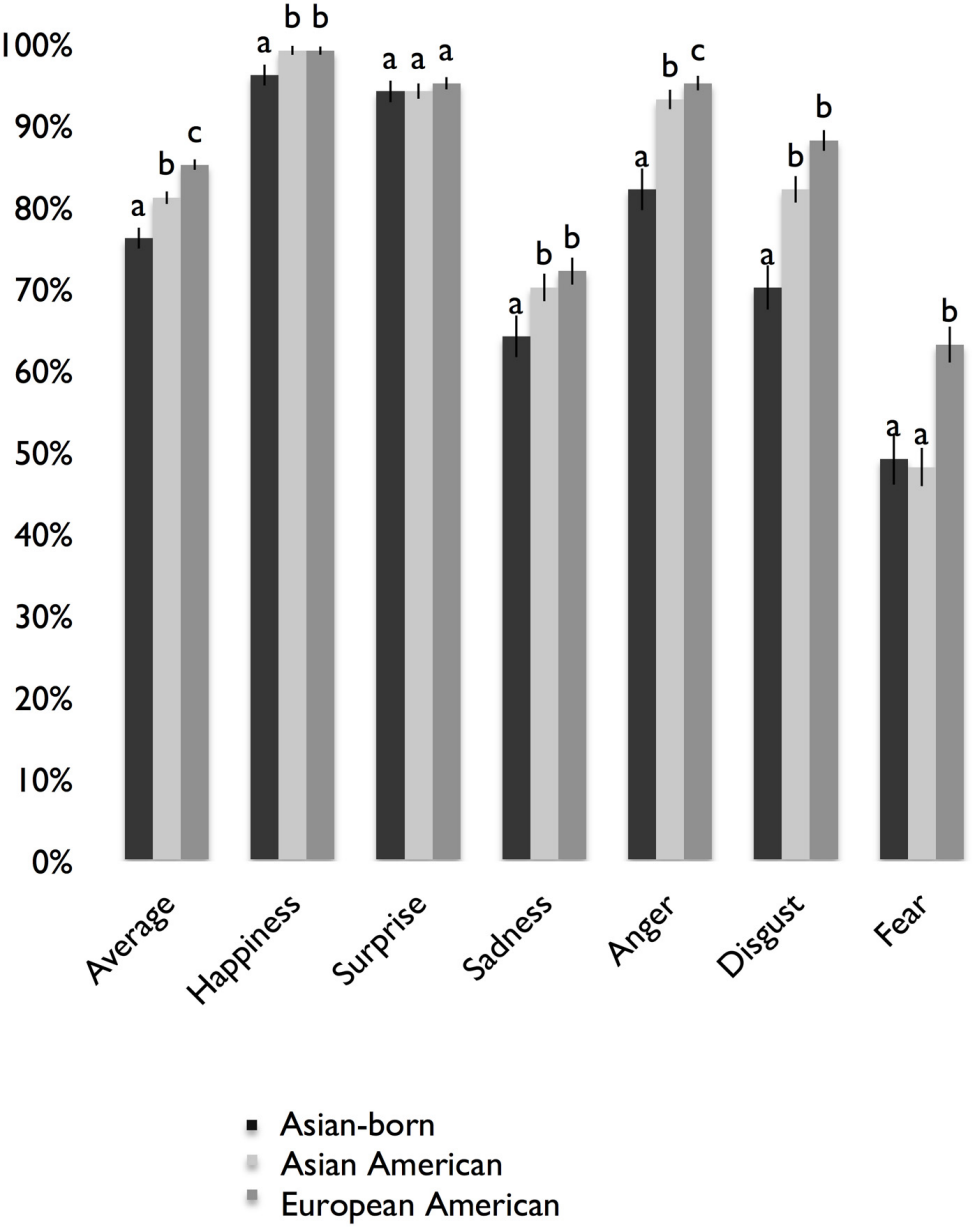
Mean Recognition Rates for the Basic Emotions in U.S. Sample. *N* = 482. Different letters denote significant differences in emotion recognition rates. Standard error bars are shown.

Fig 3 shows mean recognition rates (averaged across posers) for the basic emotion expressions of anger, disgust, fear, happiness, sadness, surprise, as well as the average across these emotions. Across groups, the highest recognition rates were found for happiness, followed by surprise, anger, disgust, sadness, and then fear. The recognition rates in the total sample were significantly greater than chance for all emotions (*p* < .05), based on binomial tests with chance set at 8% (based on the number of distinct emotion categories). As in Study 1, we also conducted binomial tests using the more stringent rate of 25%, finding that the recognition rate for shame was no longer significantly greater than chance for Asian-born participants. There were significant effects of group on emotion recognition rates. As Table 6 shows, regarding expressions of the basic emotions, there were differences among all three groups in the recognition of disgust; differences between the American groups and Asian-born group in the recognition of anger, happiness, and sadness; and there were differences between the European American and Asian groups (Asian American and Asian-born group) in the recognition of fear. There were no differences among the groups in the recognition of surprise.

**Table 6:** Regressions Examining Effect of Cultural Group on Recognition Rates for the Basic Emotions in U.S. Sample.

Model	*B*	*SE B*	*β*	*R* ^*2*^
Average				.10*
Asian-born	-.03*	.004	-.35	
Asian American	-.02*	.01	-.19	
Happiness				.02*
Asian-born	-.01*	.004	-.14	
Asian American	-.00004	.004	-.004	
Surprise				.002
Asian-born	-.004	.01	-.04	
Asian American	-.004	.01	-.03	
Sadness				.02*
Asian-born	-.03*	.01	-.14	
Asian American	-.01	.01	-.05	
Anger				.07*
Asian-born	-.04*	.01	-.29	
Asian American	-.01	.01	-.07	
Disgust				.09*
Asian-born	-.06*	.01	-.33	
Asian American	-.03*	.01	-.14	
Fear				.05*
Asian-born	-.05*	.01	-.17	
Asian American	-.07*	.02	-.22	

*N* = 482. Group predictors were dummy coded so that European Americans were the reference group.

* p < .05 *N* = 482. Subscripts denote significant group differences, *p* < .05.

#### Recognition of the self-conscious emotions

**Fig 4:**
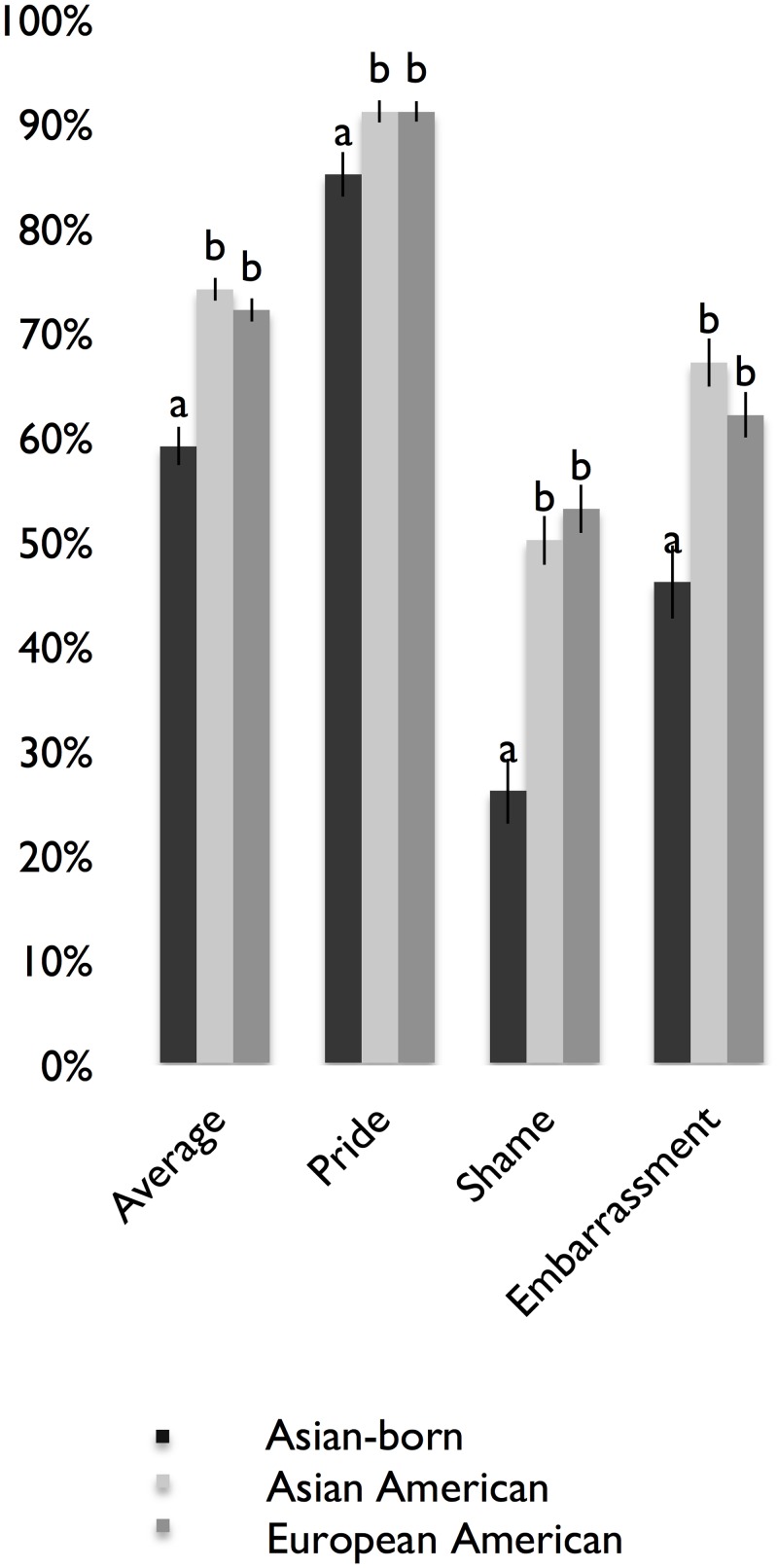
Mean Recognition Rates for the Self-Conscious Emotions in U.S. Sample. *N* = 482. Different letters denote significant differences in emotion recognition rates. Standard error bars are shown.

Fig 4 shows mean recognition rates (averaged across posers) for the self-conscious emotion expressions of embarrassment, pride, and shame, as well as the average across these expressions. Across groups, the highest recognition rates were found for pride, followed by embarrassment, and then shame. Similar to the results for the basic emotions, the recognition rates in the total sample were significantly greater than chance for all emotions (*p* < .05), and there were significant effects of group on emotion recognition rates. As Table 7 shows, regarding expressions of the self-conscious emotions of embarrassment, pride, and shame, the American groups (i.e., European Americans and Asian Americans) had higher emotion recognition rates than the Asian-born group.

**Table 7:** Regressions Examining Effect of Cultural Group on Recognition Rates for the Self-Conscious Emotions in U.S. Sample.

Model	*B*	*SE B*	*β*	*R* ^*2*^
Average				.11*
Asian-born	-.04*	.01	-.31	
Asian American	.01	.01	.03	
Pride				.02*
Asian-born	-.02*	.01	-.13	
Asian American	.002	.01	.01	
Shame				.09*
Asian-born	-.09*	.01	-.32	
Asian American	-.01	.02	-.04	
Embarrassment				.06*
Asian-born	-.05*	.01	-.20	
Asian American	.02	.02	.07	

*N* = 482. Group predictors were dummy coded so that European Americans were the reference group.

* p < .05

#### Group differences in cultural values

There were also significant effects of group on cultural values. In general, the effects were in the expected directions. As Table 8 shows, the Asian-born group endorsed conformity to norms and emotional self-control more than Asian Americans, who in turn endorsed these values more than the European Americans. The Asian groups endorsed collectivism and family recognition through achievement more than the European Americans; there were no differences between Asian Americans and the Asian-born group. Contrary to our predictions, there were no significant differences among the groups on humility. Asian Americans reported being more concerned with loss of face than both the European Americans and the Asian-born group; there were no differences between the European Americans and the Asian-born group.

**Table 8:** Regressions Examining Effect of Cultural Group on Cultural Values for U.S. Sample.

Model	*B*	*SE B*	*β*	*R* ^*2*^
Collectivism				.01
Asian-born	.06	.03	.09	
Asian American	.08*	.04	.10	
Conformity to Norms				.08*
Asian-born	.24*	.04	.31	
Asian American	.13*	.05	.14	
Emotional Self-Control				.07*
Asian-born	.20*	.03	.29	
Asian American	.15*	.04	.17	
Family Recognition Through Achievement				.05*
Asian-born	.10*	.04	.14	
Asian American	.22*	.04	.24	
Humility				.001
Asian-born	.02	.04	.03	
Asian American	.001	.04	.001	
Loss of Face				.02*
Asian-born	-.0003	.03	-.0005	
Asian American	.11*	.04	.13	

*N* = 482. Group predictors were dummy coded so that European Americans were the reference group.

* p < .05.

#### Exploring the contribution of cultural values to group differences in emotion recognition rates

To examine whether the cultural values might be contributing to the variability we found in emotion recognition rates, we ran a series of hierarchical regressions, entering each of the cultural variables (separately for each variable) in the first step, and then cultural group in the second step, with emotion recognition rates (separately for each emotion) as the outcome. We tested the following recognition rates for the self-conscious emotions: pride, shame, embarrassment, and the average of these; we tested the following recognition rates for the basic emotions: happiness, sadness, anger, disgust, fear, happiness, and the average of these. The cultural values tested were: collectivism, conformity to norms, emotional self-control, family recognition through achievement, and loss of face. Because we did not find significant group differences in the recognition of surprise and in endorsement of humility, we did not examine them further.

When looked at separately, the cultural variables of collectivism, conformity to norms, emotional self-control, family recognition through achievement, and loss of face did not eliminate group differences in emotion recognition rates for the basic emotions, nor for the self-conscious emotions, as shown in Tables 3 and 4 in the Appendix. To test whether the cultural values combined might contribute to differences in emotion recognition, we entered collectivism, conformity to norms, emotional-self-control, family recognition through achievement, and loss of face as simultaneous covariates (as shown in Tables 9 and 10), and we found that the differences between the European Americans and Asian groups were no longer significant for the recognition of pride. We then looked at change in variance explained from Step 1 to Step 2 to gauge the contribution of cultural values relative to that of cultural group, and found that in general, variability in emotion recognition was not substantially explained by cultural values, relative to that was explained by cultural group. Regarding the basic emotions, change in variance explained from Step 1 to Step 2 increased by at least two times for anger, disgust, and fear. There were no significant effects of cultural variables on recognition rates for happiness and sadness, although the variance explained increased twice as much when the group variable was entered into the model. Regarding the self-conscious emotions, change in variance explained from Step 1 to Step 2 increased by at least three times, with the exception of pride.

**Table 9:** Hierarchical Regressions on Recognition of the Basic Emotions for U.S. Sample with All Cultural Variables Entered in the First Step, and Cultural Group Entered in the Second Step.

Emotion	Model	*B*	*SE B*	*β*	*R* ^*2*^	*ΔR* ^*2*^
Basic Emotions	Step 1				.04*	
	Collectivism	.003	.01	.02		
	Conformity to Norms	-.02*	.01	-.17		
	Emotional Self-Control	-.01	.01	-.09		
	Family Recognition Through Achievement	.01	.01	.07		
	Loss of Face	.01*	.01	.11		
	Step 2				.12*	.08*
	Collectivism	.002	.01	.02		
	Conformity to Norms	-.01*	.01	-.11		
	Emotional Self-Control	-.004	.01	-.03		
	Family Recognition Through Achievement	.01*	.01	.10		
	Loss of Face	.01	.01	.09		
	Asian-born	-.03*	.01	-.32		
	Asian American	-.02*	.01	-.21		
Happiness	Step 1				.01	
	Collectivism	.001	.01	.01		
	Conformity to Norms	-.01	.01	-.06		
	Emotional Self-Control	-.01	.01	-.06		
	Family Recognition Through Achievement	.001	.01	.01		
	Loss of Face	.01	.01	.06		
	Step 2				.02	.01
	Collectivism	.0001	.01	.001		
	Conformity to Norms	-.003	.01	-.03		
	Emotional Self-Control	-.004	.01	-.04		
	Family Recognition Through Achievement	.001	.01	.01		
	Loss of Face	.004	.01	.04		
	Asian-born	-.01*	.004	-.12		
	Asian American	.00005	.01	.001		
Sadness	Step 1				.01	
	Collectivism	-.003	.02	-.01		
	Conformity to Norms	.02	.01	.06		
	Emotional Self-Control	-.01	.01	-.04		
	Family Recognition Through Achievement	-.0002	.01	-.001		
	Loss of Face	.02	.01	.08		
	Step 2				.03*	.02*
	Collectivism	-.004	.01	-.02		
	Conformity to Norms	.03	.01	.10		
	Emotional Self-Control	-.004	.01	-.01		
	Family Recognition Through Achievement	.001	.01	.01		
	Loss of Face	.02	.01	.06		
	Asian-born	-.03*	.01	-.16		
	Asian American	-.02	.01	-.07		
Anger	Step 1				.04*	
	Collectivism	.01	.01	.03		
	Conformity to Norms	-.03*	.01	-.14		
	Emotional Self-Control	-.02	.01	-.08		
	Family Recognition Through Achievement	.02*	.01	.11		
	Loss of Face	.02*	.01	.10		
	Step 2				.09*	.06*
	Collectivism	.004	.01	.02		
	Conformity to Norms	-.02	.01	-.08		
	Emotional Self-Control	-.01	.01	-.03		
	Family Recognition Through Achievement	.02*	.01	.12		
	Loss of Face	.02	.01	.07		
	Asian-born	-.04*	.01	-.27		
	Asian American	-.02	.01	-.09		
Disgust	Step 1				.06*	
	Collectivism	-.01	.01	-.04		
	Conformity to Norms	-.05*	.01	-.21		
	Emotional Self-Control	-.03	.01	-.09		
	Family Recognition Through Achievement	.03*	.01	.13		
	Loss of Face	.02	.01	.06		
	Step 2				.12*	.06*
	Collectivism	-.01	.01	-.04		
	Conformity to Norms	-.04*	.01	-.15		
	Emotional Self-Control	-.01	.01	-.04		
	Family Recognition Through Achievement	.04*	.01	.14		
	Loss of Face	.01	.01	.03		
	Asian-born	-.05*	.01	-.29		
	Asian American	-.04*	.01	-.15		
Fear	Step 1				.02	
	Collectivism	.03	.02	.07		
	Conformity to Norms	-.05*	.02	-.14		
	Emotional Self-Control	-.01	.02	-.02		
	Family Recognition Through Achievement	-.01	.02	-.04		
	Loss of Face	.01	.02	.03		
	Step 2				.06*	.04*
	Collectivism	.03	.02	.07		
	Conformity to Norms	-.04*	.02	-.12		
	Emotional Self-Control	.01	.02	.01		
	Family Recognition Through Achievement	-.0002	.02	-.001		
	Loss of Face	.01	.02	.04		
	Asian-born	-.04*	.01	-.14		
	Asian American	-.07*	.02	-.22		

*N* = 482. Group predictors were dummy coded so that European Americans were the reference group.

* *p* < .05

**Table 10:** Hierarchical Regressions on Recognition of the Self-Conscious Emotions for U.S. Sample with All Cultural Variables Entered in the First Step, and Cultural Group Entered in the Second Step.

Emotion	Model	*B*	*SE B*	*β*	*R* ^*2*^	*ΔR* ^*2*^
Self-Conscious Emotions	Step 1				.04*	
	Collectivism	-.003	.01	-.02		
	Conformity to Norms	-.03*	.01	-.16		
	Emotional Self-Control	-.01	.01	-.05		
	Family Recognition Through Achievement	.01	.01	.07		
	Loss of Face	.03*	.01	.15		
	Step 2				.12*	.08*
	Collectivism	-.01	.01	-.03		
	Conformity to Norms	-.02	.01	-.09		
	Emotional Self-Control	-.001	.01	-.01		
	Family Recognition Through Achievement	.01	.01	.05		
	Loss of Face	.02*	.01	.11		
	Asian-born	-.04*	.01	-.29		
	Asian American	.004	.01	.02		
Pride	Step 1				.03*	
	Collectivism	-.01	.01	-.04		
	Conformity to Norms	-.01	.01	-.05		
	Emotional Self-Control	-.02*	.01	-.13		
	Family Recognition Through Achievement	.002	.01	.01		
	Loss of Face	.02*	.01	.11		
	Step 2				.04*	.01
	Collectivism	-.01	.01	-.04		
	Conformity to Norms	-.01	.01	-.03		
	Emotional Self-Control	-.02*	.01	-.11		
	Family Recognition Through Achievement	.0004	.01	.002		
	Loss of Face	.02	.01	.10		
	Asian-born	-.01	.01	-.09		
	Asian American	.004	.008	.03		
Shame	Step 1				.03*	
	Collectivism	-.01	.02	-.01		
	Conformity to Norms	-.05*	.02	-.14		
	Emotional Self-Control	-.002	.02	-.01		
	Family Recognition Through Achievement	.03	.02	.07		
	Loss of Face	.04*	.02	.10		
	Step 2				.10*	.08*
	Collectivism	-.01	.02	-.03		
	Conformity to Norms	-.03	.02	-.07		
	Emotional Self-Control	.02	.02	.05		
	Family Recognition Through Achievement	.03	.02	.07		
	Loss of Face	.03	.02	.06		
	Asian-born	-.09*	.01	-.32		
	Asian American	-.02	.02	-.06		
Embarrassment	Step 1				.02*	
	Collectivism	.01	.02	.03		
	Conformity to Norms	-.05*	.02	-.14		
	Emotional Self-Control	-.01	.02	-.03		
	Family Recognition Through Achievement	.01	.02	.04		
	Loss of Face	.04*	.02	.10		
	Step 2				.06*	.04*
	Collectivism	.01	.02	.02		
	Conformity to Norms	-.03	.02	-.09		
	Emotional Self-Control	.0001	.02	.0003		
	Family Recognition Through Achievement	.01	.02	.02		
	Loss of Face	.03	.02	.07		
	Asian-born	-.05*	.01	-.17		
	Asian American	.02	.02	.07		

*N* = 482. Group predictors were dummy coded so that European Americans were the reference group.

* *p* < .05

### Discussion

Study 2 replicates and extends previous literature on the recognition of the self-conscious emotions and on endorsement of Asian cultural values. Study 2 also examines the explanatory role of cultural values in the recognition of the self-conscious emotions. Several findings are particularly noteworthy.

First, the findings replicate previous research on the recognition of the self-conscious emotions, and also, suggest that even within the United States, that there is cultural variability in the recognition of both the basic and self-conscious emotions. Regarding the basic emotions, the group differences in recognition rates for anger and happiness were similar to those found for the self-conscious emotions. For the recognition of disgust, European Americans had higher recognition rates than Asian Americans, who in turn had higher recognition rates than Asian-born individuals. For fear, European Americans had higher recognition rates than Asian Americans, who had similar recognition rates to the Asian-born individuals. However, we found no group differences in recognition of the expression of surprise. Regarding the self-conscious emotions, European Americans and Asian Americans showed similar and higher recognition rates than Asian-born individuals.

Second, the findings also replicate previous research on Asian cultural values within the United States [41][37]. We found that cultural values of conformity to norms and emotional self control were endorsed the most by Asian-born individuals, then by Asian Americans, and least by European Americans; cultural values of collectivism and family recognition through achievement were endorsed the most and similarly by Asian-born participants and Asian Americans, and least by European Americans. However, we did not find differences in humility, and the differences found for loss of face were counter to previous theory and research [38]. We found that Asian Americans were highest in face concerns, while Asian-born individuals and European Americans were less so and similar to each other. We would expect that the Asian-born individuals would be most concerned with loss of face, but it could be that this difference might speak to something unique about the Asian-born individuals in our sample.

Third, although in general, we found significant variability in the cultural values we measured, we found that they only fully explained group differences in recognition of pride, and this was due collectively from differential endorsement of the cultural values of collectivism, conformity to norms, emotional self-control, and family recognition through achievement for recognition of pride. When we looked to variance explained for a more nuanced explanation, we found that cultural variables accounted for far less variance on their own in comparison to when group effects were also considered, with the exception of pride. While these results are interesting and informative, they do not clearly support the Cultural Values Hypothesis. One possibility is that we measured cultural values using measures of actual self-importance (i.e., how personally important the value is to a member of a cultural group) rather than measures of perceived cultural importance (i.e., how important the value is believed to be to the culture) [42]. Future research is recommended to examine whether measures of perceived cultural importance might explain more than what the present research has found.

Fourth, we found indirect evidence for the Exposure Hypothesis, which would expect that emotion recognition rates would be variable depending on exposure to Western culture. Therefore, we expected that there would be no differences in emotion recognition rates between the European Americans and Asian Americans, because their level of exposure would be the same; we expected there to be significant differences in emotion recognition rates between the two American groups and the Asian-born group, expecting lower recognition rates for Asian-born individuals because of lower exposure to Western culture. In general, our results were consistent with the Exposure Hypothesis. Differences in emotion recognition were similar between the European American and Asian American groups, and higher than those for Asian-born individuals for recognition of the basic emotions of anger and happiness, and for the self-conscious emotions of embarrassment, pride, and shame (i.e., five of eight of the emotions whose recognition rates were different across groups).

In summary, Study 2 contributes to the small literature on the recognition of self-conscious emotions by not only replicating and extending previous findings, but also exploring how cultural values and exposure to Western culture might explain differences in emotion recognition.

## General Discussion

Previous research suggests that the self-conscious emotions of embarrassment, shame, and pride have distinct, nonverbal expressions that can be recognized in the United States at above-chance levels. However, few studies have examined the recognition of these emotions in Asia. Study 1 examined recognition of the self-conscious emotion expressions in South Korea and found that recognition rates were very high for pride, low but above chance for shame, and near zero for embarrassment. Across a range of basic and self-conscious emotions, our sample of South Koreans showed lower recognition rates to rates previously found in the U.S., but were comparable to non-Western cultures that have been previously studied.

Study 2 sought to examine what might help us understand the relatively low recognition rates we found in South Korea. Such factors as exposure to Western culture and cultural values have been identified as possible contributors to cultural variability in recognition rates for the basic emotions. Recognition of basic emotions, self-conscious emotions, and endorsement of several cultural values were examined in a sample of U.S. college students that differed both in exposure to Western culture and endorsement of Asian cultural values (i.e., European American, Asian American, and Asian-born individuals). Emotion recognition rates were largely similar between the European Americans and Asian Americans and higher than for the Asian-born individuals. We reasoned that cultural values might explain differences in the recognition of the self-conscious emotions for these groups because of previous theorizing, prior research, and findings from the present research that there were cultural differences in both emotion recognition rates and endorsement of cultural values. We found that differences in emotion recognition for pride were no longer statistically significant when we accounted for variability in cultural values, and that some variance was explained by cultural values, but the majority of the variability in recognition rates was explained by group differences. While these findings are interesting, these findings do not neatly reflect previous theorizing and research.

The most parsimonious explanation we can generate from the present research is that it is exposure to Western culture, and not cultural values that are driving differences in recognition of the self-conscious emotions. The primary evidence for this is our finding that emotion recognition rates were similar between the European American and Asian American groups, but higher than for the Asian-born participants. We also found low recognition rates across emotion expressions in South Korea, which, when contrasted to the rates in our U.S. sample, generally support the Exposure Hypothesis.

### Limitations and future directions

The present research was not without limitation. First, although it was not the primary aim of Study 1 to do so, we did not measure cultural variables in South Korea, which did not allow us to examine potential variability within the South Korean sample, as well as between countries (e.g., between our South Korean and American samples) that would help to explain emotion recognition rates. We encourage future research assessing culture proxies.

Second, although Study 2 was intended to look at potential explanations of what might be contributing to the lower recognition rates we generally found in South Korea, we did not examine other countries in Asia, and within our U.S. sample, we did not differentiate among various Asian ethnic groups due to inadequate sample sizes. Both of these issues limit the generality of our findings. Although we examined South Korea because it is an Asian culture, we cannot assume that our findings generalize to all Asian cultures, as others have argued [43]. Furthermore, we are unsure of how representative the recent immigrants from Asia in our U.S. sample are, as there might be something unique to this group that we did not measure and that might influence their emotion recognition rates. Relatedly, it is important interpret the findings from both studies under the caveat that our samples consisted solely of university students, raising the possibility that we might have found very different emotion recognition rates if we had conducted the study within community samples. We encourage future research that examines emotion recognition of the self-conscious emotions across a range of Asian cultures, Asian ethnicities within U.S. samples, and in non-university samples.

Third, we were unable to study factors such as the ingroup advantage, and cultural differences in cognition. One of the reasons for not testing the ingroup advantage was that we did not have a stimulus set of nonverbal expressions that had Asian posers expressing pride, shame, and embarrassment. Additionally, several recent studies suggest that the East Asian perceivers may show distinct differences in the way they recognize emotions; for example, they rely more on social contextual cues [44], may focus more on the eyes (rather than the eyes and mouth as their Western counterparts do) when judging emotion expressions [45], and are more attuned to vocal cues in the presence of incongruent facial expressions [46]. Future research should examine how these factors might relate to the recognition to the self-conscious emotions.

Fourth, our findings suggest that exposure to Western culture may be explaining cultural differences in emotion recognition rates and this interpretation is consistent with previous literature [28]. One suggestion for future research is to examine expressions of emotion posed by Asian expressers from existing sets on Asian participants of varying levels of acculturation (e.g., Montreal Set of Facial Displays of Emotion [47]; Japanese and Caucasian Facial Expressions of Emotion [48]). By using a stimulus set that has Asian posers, exposure to North American culture can be contrasted to exposure to targets. Intriguingly, a recent study examining emotion recognition accuracy among Caucasian Australians, Chinese Australians, and Mainland Chinese found that acculturation into Australian culture contributed modestly to differences in emotion recognition rates for expressions posed by Caucasians [49], but exposure (i.e., length of time in Australia) did not, suggesting that increased engagement with Australian culture was associated with higher accuracy. However, in another recent study [50], researchers found that greater frequency of emotion expression (i.e., how often the emotion expression was encountered in daily life) was strongly and positively associated with emotion recognition accuracy, suggesting that exposure to the actual expression itself is integral to accuracy. Further research is needed to examine these issues.

### Conclusion

Overall, the present findings provide insights into the cross-cultural recognition of the self-conscious emotions of pride, shame, and embarrassment. We found further evidence for the cross-cultural generality of the nonverbal expressions of pride and shame in South Korea. We then replicated these findings in an U.S. college student sample that included recent immigrants from Asia, Asian Americans, and European Americans, and found significant variability in recognition of the self-conscious and basic emotions across these groups. These differences in emotion recognition rates did not disappear in a meaningful way when controlling for cultural values, suggesting that exposure to Western culture is a more important for emotion recognition than values.

### Ethics statement

Approval for this research was obtained from the Institutional Review Board at the University of California, Davis. Participation was voluntary, and written consent was obtained before the study began.

## Supporting Information

S1 Appendix Appendix. (DOCX)

S1 Dataset Study 1.sav. SPSS data file for Study 1.
(SAV)

S2 Dataset Study 2.sav. SPSS data file for Study 2.
(SAV)

S1 Syntax Study 1.sps. SPSS syntax file for Study 1.
(SPS)

S2 Syntax Study 2.sps. SPSS syntax file for Study 2.
(SPS)
