# Supplementary material for: Exploring Cultural Differences in the Recognition of the Self-Conscious Emotions
Source: PLoS One. 2015 Aug 26;10(8):e0136411. doi: 10.1371/journal.pone.0136411 (PMC4550404; doi:10.1371/journal.pone.0136411)
Supplement: S1 Appendix — (DOCX) [file pone.0136411.s001.docx]

**Table 1. Demographic Information for Asian-born Participants in the U.S. Sample**

|  |  |  |  |  |
| --- | --- | --- | --- | --- |
|  | Demographic Variable |  |  |  |
|  |  |  |  |  |
|  | Years in United States |  |  |  |
|  | *M* |  | 5.03 |  |
|  | *SD* |  | 3.44 |  |
|  | Country of origin |  |  |  |
|  | China |  | 31% |  |
|  | South Korea |  | 27% |  |
|  | Japan |  | 9% |  |
|  | Taiwan |  | 6% |  |
|  | Hong Kong |  | 5% |  |
|  | India |  | 5% |  |
|  | Vietnam |  | 5% |  |
|  | Philippines |  | 4% |  |
|  | Indonesia |  | 2% |  |
|  | Pakistan |  | 2% |  |
|  | Fiji Islands |  | 1% |  |
|  | Guam |  | 1% |  |
|  | Nepal |  | 1% |  |
|  | Native language |  |  |  |
|  | Chinese |  | 27% |  |
|  | Korean |  | 26% |  |
|  | Cantonese |  | 10% |  |
|  | Japanese |  | 8% |  |
|  | Mandarin |  | 7% |  |
|  | Vietnamese |  | 5% |  |
|  | Tagalog |  | 3% |  |
|  | English |  | 2% |  |
|  | Hindi |  | 2% |  |
|  | Punjabi |  | 2% |  |
|  | Urdu |  | 2% |  |
|  | Gujarati |  | 1% |  |
|  | Indonesian |  | 1% |  |
|  | Nepalese |  | 1% |  |
|  | Visayan |  | 1% |  |
|  |  |  |  |  |

*N* = 96.

**Table 2. Intercorrelations among Cultural Variables for U.S. Sample by Cultural Group**

|  |  |  |  |  |  |  |  |  |
| --- | --- | --- | --- | --- | --- | --- | --- | --- |
|  | Cultural Group |  | 1 | 2 | 3 | 4 | 5 |  |
|  |  |  |  |  |  |  |  |  |
|  | Asian-born |  |  |  |  |  |  |  |
|  | 1. Collectivism |  |  |  |  |  |  |  |
|  | 2. Conformity to Norms |  | .42* |  |  |  |  |  |
|  | 3. Emotional Self-Control |  | .27* | .36* |  |  |  |  |
|  | 4. Family Recognition through Achievement |  | .40* | .50* | .34* |  |  |  |
|  | 5. Humility |  | .16 | .03 | .28* | .05 |  |  |
|  | 6. Loss of Face |  | .22* | .27* | .32* | .22* | .11 |  |
|  | Asian American |  |  |  |  |  |  |  |
|  | 1. Collectivism |  |  |  |  |  |  |  |
|  | 2. Conformity to Norms |  | .24* |  |  |  |  |  |
|  | 3. Emotional Self-Control |  | .18* | .33* |  |  |  |  |
|  | 4. Family Recognition through Achievement |  | .28* | .24* | .22* |  |  |  |
|  | 5. Humility |  | .16* | -.07 | .22* | -.03 |  |  |
|  | 6. Loss of Face |  | .20* | .33* | .17* | .27* | -.02 |  |
|  | European American |  |  |  |  |  |  |  |
|  | 1. Collectivism |  |  |  |  |  |  |  |
|  | 2. Conformity to Norms |  | .25* |  |  |  |  |  |
|  | 3. Emotional Self-Control |  | .24* | .31* |  |  |  |  |
|  | 4. Family Recognition through Achievement |  | .15* | .31* | .14* |  |  |  |
|  | 5. Humility |  | .16* | -.13 | .19* | -.01 |  |  |
|  | 6. Loss of Face |  | .05 | .21* | .19* | .16* | .19* |  |
|  |  |  |  |  |  |  |  |  |

* *p* < .05

**Table 3. Hierarchical Regressions on Recognition of the Basic Emotions for U.S. Sample with All Cultural Variables Entered Separately in the First Step, and Cultural Group Entered in the Second Step**

|  |  |  |  |  |  |  |  |  |  |  |
| --- | --- | --- | --- | --- | --- | --- | --- | --- | --- | --- |
|  | Emotion | Model |  |  | *B* | *SE B* | *β* | *R^2^* | *∆R^2^* |  |
|  |  |  |  |  |  |  |  |  |  |  |
|  | Basic Emotions | Step 1 | Collectivism |  | -.002 | .01 | -.01 | .0002 |  |  |
|  |  | Step 2 | Collectivism |  | .002 | .01 | .01 | .10* | .10* |  |
|  |  |  | Recent Immigrant from Asia |  | -.03* | .004 | -.35 |  |  |  |
|  |  |  | Asian American |  | -.02* | .01 | -.19 |  |  |  |
|  |  | Step 1 | Conformity to Norms |  | -.02* | .01 | -.15 | .02* |  |  |
|  |  | Step 2 | Conformity to Norms |  | -.01 | .01 | -.06 | .11* | .08* |  |
|  |  |  | Recent Immigrant from Asia |  | -.03* | .01 | -.33 |  |  |  |
|  |  |  | Asian American |  | -.02* | .01 | -.18 |  |  |  |
|  |  | Step 1 | Emotional Self-Control |  | -.01* | .01 | -.11 | .012* |  |  |
|  |  | Step 2 | Emotional Self-Control |  | -.003 | .01 | -.03 | .10* | .09* |  |
|  |  |  | Recent Immigrant from Asia |  | -.03* | .01 | -.34 |  |  |  |
|  |  |  | Asian American |  | -.02* | .01 | -.19 |  |  |  |
|  |  | Step 1 | Family Recognition through Achievement |  | .003 | .01 | .02 | .001 |  |  |
|  |  | Step 2 | Family Recognition through Achievement |  | .01 | .01 | .08 | .11* | .11* |  |
|  |  |  | Recent Immigrant from Asia |  | -.03* | .004 | -.36 |  |  |  |
|  |  |  | Asian American |  | -.02* | .01 | -.21 |  |  |  |
|  |  | Step 1 | Loss of Face |  | .01 | .01 | .07 | .004 |  |  |
|  |  | Step 2 | Loss of Face |  | .01 | .01 | .08 | .11* | .10* |  |
|  |  |  | Recent Immigrant from Asia |  | -.03* | .004 | -.34 |  |  |  |
|  |  |  | Asian American |  | -.02* | .01 | -.20 |  |  |  |
|  | Happiness | Step 1 | Collectivism |  | -.002 | .01 | -.02 | .0003 |  |  |
|  |  | Step 2 | Collectivism |  | -.001 | .01 | -.01 | .02* | .02* |  |
|  |  |  | Recent Immigrant from Asia |  | -.01* | .004 | -.13 |  |  |  |
|  |  |  | Asian American |  | -.0003 | .004 | -.003 |  |  |  |
|  |  | Step 1 | Conformity to Norms |  | -.01 | .004 | -.06 | .004 |  |  |
|  |  | Step 2 | Conformity to Norms |  | -.003 | .01 | -.03 | .02* | .02* |  |
|  |  |  | Recent Immigrant from Asia |  | -.01* | .004 | -.13 |  |  |  |
|  |  |  | Asian American |  | .00003 | .004 | .0004 |  |  |  |
|  |  | Step 1 | Emotional Self-Control |  | -.01 | .01 | -.07 | .01 |  |  |
|  |  | Step 2 | Emotional Self-Control |  | -.004 | .01 | -.04 | .02* | .02* |  |
|  |  |  | Recent Immigrant from Asia |  | -.01* | .004 | -.12 |  |  |  |
|  |  |  | Asian American |  | .0003 | .004 | .003 |  |  |  |
|  |  | Step 1 | Family Recognition through Achievement |  | -.001 | .01 | -.01 | .0001 |  |  |
|  |  | Step 2 | Family Recognition through Achievement |  | .00008 | .01 | -.001 | .02* | .02* |  |
|  |  |  | Recent Immigrant from Asia |  | -.01* | .004 | -.13 |  |  |  |
|  |  |  | Asian American |  | -.0003 | .01 | -.004 |  |  |  |
|  |  | Step 1 | Loss of Face |  | .003 | .01 | .03 | .001 |  |  |
|  |  | Step 2 | Loss of Face |  | .003 | .01 | .03 | .02* | .02* |  |
|  |  |  | Recent Immigrant from Asia |  | -.01* | .004 | -.14 |  |  |  |
|  |  |  | Asian American |  | -.001 | .004 | -.01 |  |  |  |
|  | Sadness | Step 1 | Collectivism |  | .002 | .01 | .01 | .0001 |  |  |
|  |  | Step 2 | Collectivism |  | .01 | .01 | .02 | .02 | .02* |  |
|  |  |  | Recent Immigrant from Asia |  | -.03* | .01 | -.14 |  |  |  |
|  |  |  | Asian American |  | -.01 | .01 | -.05 |  |  |  |
|  |  | Step 1 | Conformity to Norms |  | .02 | .01 | .07 | .004 |  |  |
|  |  | Step 2 | Conformity to Norms |  | .03* | .01 | .11 | .03* | .02* |  |
|  |  |  | Recent Immigrant from Asia |  | -.03* | .01 | -.17 |  |  |  |
|  |  |  | Asian American |  | -.02 | .01 | -.06 |  |  |  |
|  |  | Step 1 | Emotional Self-Control |  | -.001 | .01 | -.004 | .00002 |  |  |
|  |  | Step 2 | Emotional Self-Control |  | .01 | .01 | .03 | .02 | .02* |  |
|  |  |  | Recent Immigrant from Asia |  | -.03* | .01 | -.14 |  |  |  |
|  |  |  | Asian American |  | -.01 | .01 | -.05 |  |  |  |
|  |  | Step 1 | Family Recognition through Achievement |  | .01 | .01 | .03 | .001 |  |  |
|  |  | Step 2 | Family Recognition through Achievement |  | .01 | .01 | .04 | .02* | .02* |  |
|  |  |  | Recent Immigrant from Asia |  | -.03* | .01 | -.14 |  |  |  |
|  |  |  | Asian American |  | -.01 | .01 | -.06 |  |  |  |
|  |  | Step 1 | Loss of Face |  | .02 | .01 | .09 | .01 |  |  |
|  |  | Step 2 | Loss of Face |  | .03 | .01 | .09 | .02* | .02* |  |
|  |  |  | Recent Immigrant from Asia |  | -.03* | .01 | -.14 |  |  |  |
|  |  |  | Asian American |  | -.01 | .01 | -.06 |  |  |  |
|  | Anger | Step 1 | Collectivism |  | .002 | .01 | .01 | .00009 |  |  |
|  |  | Step 2 | Collectivism |  | .01 | .01 | .03 | .07* | .07* |  |
|  |  |  | Recent Immigrant from Asia |  | -.04* | .01 | -.29 |  |  |  |
|  |  |  | Asian American |  | -.01 | .01 | -.07 |  |  |  |
|  |  | Step 1 | Conformity to Norms |  | -.02* | .01 | -.10 | .01* |  |  |
|  |  | Step 2 | Conformity to Norms |  | -.004 | .01 | -.02 | .07* | .06* |  |
|  |  |  | Recent Immigrant from Asia |  | -.04* | .01 | -.28 |  |  |  |
|  |  |  | Asian American |  | -.01 | .01 | -.07 |  |  |  |
|  |  | Step 1 | Emotional Self-Control |  | -.02 | .01 | -.08 | .01 |  |  |
|  |  | Step 2 | Emotional Self-Control |  | -.003 | .01 | -.01 | .07* | .07* |  |
|  |  |  | Recent Immigrant from Asia |  | -.04* | .01 | -.28 |  |  |  |
|  |  |  | Asian American |  | -.01 | .01 | -.07 |  |  |  |
|  |  | Step 1 | Family Recognition through Achievement |  | .02 | .01 | .08 | .01 |  |  |
|  |  | Step 2 | Family Recognition through Achievement |  | .02* | .01 | .11 | .08* | .08* |  |
|  |  |  | Recent Immigrant from Asia |  | -.04* | .01 | -.30 |  |  |  |
|  |  |  | Asian American |  | -.02 | .01 | -.10 |  |  |  |
|  |  | Step 1 | Loss of Face |  | .02 | .01 | .08 | .01 |  |  |
|  |  | Step 2 | Loss of Face |  | .02 | .01 | .08 | .08* | .07* |  |
|  |  |  | Recent Immigrant from Asia |  | -.04* | .01 | -.29 |  |  |  |
|  |  |  | Asian American |  | -.02 | .01 | -.08 |  |  |  |
|  | Disgust | Step 1 | Collectivism |  | -.02 | .01 | -.08 | .01 |  |  |
|  |  | Step 2 | Collectivism |  | -.02 | .01 | -.05 | .09* | .09* |  |
|  |  |  | Recent Immigrant from Asia |  | -.06* | .01 | -.32 |  |  |  |
|  |  |  | Asian American |  | -.03* | .01 | -.14 |  |  |  |
|  |  | Step 1 | Conformity to Norms |  | -.05* | .01 | -.20 | .04* |  |  |
|  |  | Step 2 | Conformity to Norms |  | -.03* | .01 | -.12 | .10* | .07* |  |
|  |  |  | Recent Immigrant from Asia |  | -.06* | .01 | -.29 |  |  |  |
|  |  |  | Asian American |  | -.03* | .01 | -.13 |  |  |  |
|  |  | Step 1 | Emotional Self-Control |  | -.04* | .01 | -.14 | .02* |  |  |
|  |  | Step 2 | Emotional Self-Control |  | -.02 | .01 | -.06 | .09* | .08* |  |
|  |  |  | Recent Immigrant from Asia |  | -.06* | .01 | -.31 |  |  |  |
|  |  |  | Asian American |  | -.03* | .01 | -.13 |  |  |  |
|  |  | Step 1 | Family Recognition through Achievement |  | .01 | .01 | .04 | .002 |  |  |
|  |  | Step 2 | Family Recognition through Achievement |  | .02 | .01 | .08 | .10* | .09* |  |
|  |  |  | Recent Immigrant from Asia |  | -.06* | .01 | -.34 |  |  |  |
|  |  |  | Asian American |  | -.04* | .01 | -.16 |  |  |  |
|  |  | Step 1 | Loss of Face |  | .003 | .01 | .01 | .0001 |  |  |
|  |  | Step 2 | Loss of Face |  | .004 | .01 | .01 | .09* | .09* |  |
|  |  |  | Recent Immigrant from Asia |  | -.06* | .01 | -.33 |  |  |  |
|  |  |  | Asian American |  | -.03* | .01 | -.14 |  |  |  |
|  | Fear | Step 1 | Collectivism |  | .01 | .02 | .02 | .0004 |  |  |
|  |  | Step 2 | Collectivism |  | .02 | .02 | .04 | .05* | .05* |  |
|  |  |  | Recent Immigrant from Asia |  | -.05* | .01 | -.18 |  |  |  |
|  |  |  | Asian American |  | -.08* | .02 | -.23 |  |  |  |
|  |  | Step 1 | Conformity to Norms |  | -.05* | .02 | -.13 | .02* |  |  |
|  |  | Step 2 | Conformity to Norms |  | -.03 | .02 | -.09 | .06* | .04* |  |
|  |  |  | Recent Immigrant from Asia |  | -.04* | .01 | -.15 |  |  |  |
|  |  |  | Asian American |  | -.07* | .02 | -.21 |  |  |  |
|  |  | Step 1 | Emotional Self-Control |  | -.02 | .02 | -.05 | .003 |  |  |
|  |  | Step 2 | Emotional Self-Control |  | -.001 | .02 | -.003 | .05* | .05* |  |
|  |  |  | Recent Immigrant from Asia |  | -.05* | .01 | -.17 |  |  |  |
|  |  |  | Asian American |  | -.07* | .02 | -.22 |  |  |  |
|  |  | Step 1 | Family Recognition through Achievement |  | -.02 | .02 | -.06 | .004 |  |  |
|  |  | Step 2 | Family Recognition through Achievement |  | -.004 | .02 | -.01 | .05* | .05* |  |
|  |  |  | Recent Immigrant from Asia |  | -.05* | .01 | -.17 |  |  |  |
|  |  |  | Asian American |  | -.07* | .02 | -.22 |  |  |  |
|  |  | Step 1 | Loss of Face |  | -.002 | .02 | -.004 | .00002 |  |  |
|  |  | Step 2 | Loss of Face |  | .01 | .02 | .02 | .05* | .05* |  |
|  |  |  | Recent Immigrant from Asia |  | -.05* | .01 | -.17 |  |  |  |
|  |  |  | Asian American |  | -.07* | .02 | -.23 |  |  |  |
|  |  |  |  |  |  |  |  |  |  |  |

Group predictors were dummy coded so that European Americans were the reference group.

* *p* < .05

**Table 4. Hierarchical Regressions on Recognition of the Self-Conscious Emotions for U.S. Sample with Cultural Variables Entered Separately in the First Step, and Cultural Group Entered in the Second Step**

|  |  |  |  |  |  |  |  |  |  |  |
| --- | --- | --- | --- | --- | --- | --- | --- | --- | --- | --- |
|  | Emotion | Model |  |  | *B* | *SE B* | *β* | *R^2^* | *∆R^2^* |  |
|  |  |  |  |  |  |  |  |  |  |  |
|  | Self-Conscious Emotions | Step 1 | Collectivism |  | -.007 | .01 | -.04 | .001 |  |  |
|  |  | Step 2 | Collectivism |  | -.005 | .01 | -.02 | .11* | .11* |  |
|  |  |  | Recent Immigrant from Asia |  | -.04* | .01 | -.31 |  |  |  |
|  |  |  | Asian American |  | .01 | .01 | .03 |  |  |  |
|  |  | Step 1 | Conformity to Norms |  | -.02* | .01 | -.13 | .02* |  |  |
|  |  | Step 2 | Conformity to Norms |  | -.02 | .01 | -.05 | .11* | .09* |  |
|  |  |  | Recent Immigrant from Asia |  | -.04* | .01 | -.30 |  |  |  |
|  |  |  | Asian American |  | .01 | .01 | .04 |  |  |  |
|  |  | Step 1 | Emotional Self-Control |  | -.01 | .01 | -.07 | .01 |  |  |
|  |  | Step 2 | Emotional Self-Control |  | -.001 | .01 | -.01 | .11* | .10* |  |
|  |  |  | Recent Immigrant from Asia |  | -.04* | .01 | -.31 |  |  |  |
|  |  |  | Asian American |  | .01 | .01 | .03 |  |  |  |
|  |  | Step 1 | Family Recognition through Achievement |  | .01 | .01 | .03 | .001 |  |  |
|  |  | Step 2 | Family Recognition through Achievement |  | .01 | .01 | .04 | .11* | .11* |  |
|  |  |  | Recent Immigrant from Asia |  | -.05* | .01 | -.32 |  |  |  |
|  |  |  | Asian American |  | .004 | .01 | .02 |  |  |  |
|  |  | Step 1 | Loss of Face |  | .02* | .01 | .11 | .01* |  |  |
|  |  | Step 2 | Loss of Face |  | .02* | .01 | .09 | .12* | .11* |  |
|  |  |  | Recent Immigrant from Asia |  | -.04* | .01 | -.31 |  |  |  |
|  |  |  | Asian American |  | .003 | .01 | .02 |  |  |  |
|  | Pride | Step 1 | Collectivism |  | -.01 | .01 | -.06 | .004 |  |  |
|  |  | Step 2 | Collectivism |  | -.01 | .01 | -.06 | .02* | .02* |  |
|  |  |  | Recent Immigrant from Asia |  | -.02* | .01 | -.13 |  |  |  |
|  |  |  | Asian American |  | .003 | .01 | .02 |  |  |  |
|  |  | Step 1 | Conformity to Norms |  | -.01 | .01 | -.08 | .01 |  |  |
|  |  | Step 2 | Conformity to Norms |  | -.01 | .01 | -.05 | .02* | .02* |  |
|  |  |  | Recent Immigrant from Asia |  | -.02* | .01 | -.12 |  |  |  |
|  |  |  | Asian American |  | .003 | .01 | .02 |  |  |  |
|  |  | Step 1 | Emotional Self-Control |  | -.02* | .01 | -.13 | .02* |  |  |
|  |  | Step 2 | Emotional Self-Control |  | -.02* | .01 | -.11 | .03* | .01* |  |
|  |  |  | Recent Immigrant from Asia |  | -.01* | .01 | -.10 |  |  |  |
|  |  |  | Asian American |  | .01 | .01 | .03 |  |  |  |
|  |  | Step 1 | Family Recognition through Achievement |  | -.004 | .01 | -.02 | .0004 |  |  |
|  |  | Step 2 | Family Recognition through Achievement |  | -.003 | .01 | -.02 | .02* | .02* |  |
|  |  |  | Recent Immigrant from Asia |  | -.02* | .01 | -.13 |  |  |  |
|  |  |  | Asian American |  | .003 | .01 | .02 |  |  |  |
|  |  | Step 1 | Loss of Face |  | .01 | .01 | .07 | .004 |  |  |
|  |  | Step 2 | Loss of Face |  | .01 | .01 | .06 | .02* | .02* |  |
|  |  |  | Recent Immigrant from Asia |  | -.02* | .01 | -.13 |  |  |  |
|  |  |  | Asian American |  | .001 | .01 | .01 |  |  |  |
|  | Shame | Step 1 | Collectivism |  | -.01 | .02 | -.02 | .001 |  |  |
|  |  | Step 2 | Collectivism |  | -.003 | .02 | -.01 | .09* | .09* |  |
|  |  |  | Recent Immigrant from Asia |  | -.09* | .01 | -.32 |  |  |  |
|  |  |  | Asian American |  | -.01 | .02 | -.04 |  |  |  |
|  |  | Step 1 | Conformity to Norms |  | -.04* | .02 | -.10 | .01* |  |  |
|  |  | Step 2 | Conformity to Norms |  | -.01 | .02 | -.02 | .09* | .08* |  |
|  |  |  | Recent Immigrant from Asia |  | -.09* | .01 | -.31 |  |  |  |
|  |  |  | Asian American |  | -.01 | .02 | -.04 |  |  |  |
|  |  | Step 1 | Emotional Self-Control |  | -.01 | .02 | -.03 | .001 |  |  |
|  |  | Step 2 | Emotional Self-Control |  | .02 | .02 | .05 | .10* | .09* |  |
|  |  |  | Recent Immigrant from Asia |  | -.09* | .01 | -.33 |  |  |  |
|  |  |  | Asian American |  | -.02 | .02 | -.05 |  |  |  |
|  |  | Step 1 | Family Recognition through Achievement |  | .02 | .02 | .04 | .002 |  |  |
|  |  | Step 2 | Family Recognition through Achievement |  | .03 | .02 | .07 | .10* | .10* |  |
|  |  |  | Recent Immigrant from Asia |  | -.09* | .01 | -.33 |  |  |  |
|  |  |  | Asian American |  | -.02 | .02 | -.06 |  |  |  |
|  |  | Step 1 | Loss of Face |  | .03 | .02 | .08 | .01 |  |  |
|  |  | Step 2 | Loss of Face |  | .03 | .02 | .07 | .10* | .09* |  |
|  |  |  | Recent Immigrant from Asia |  | -.09* | .01 | -.32 |  |  |  |
|  |  |  | Asian American |  | -.02 | .02 | -.05 |  |  |  |
|  | Embarrassment | Step 1 | Collectivism |  | .001 | .02 | .004 | .00001 |  |  |
|  |  | Step 2 | Collectivism |  | .003 | .02 | .01 | .05* | .05* |  |
|  |  |  | Recent Immigrant from Asia |  | -.05* | .01 | -.20 |  |  |  |
|  |  |  | Asian American |  | .02 | .02 | .07 |  |  |  |
|  |  | Step 1 | Conformity to Norms |  | -.04* | .02 | -.10 | .01* |  |  |
|  |  | Step 2 | Conformity to Norms |  | -.02 | .02 | -.06 | .06* | .05* |  |
|  |  |  | Recent Immigrant from Asia |  | -.05* | .01 | -.18 |  |  |  |
|  |  |  | Asian American |  | .03 | .02 | .08 |  |  |  |
|  |  | Step 1 | Emotional Self-Control |  | -.02 | .02 | -.04 | .002 |  |  |
|  |  | Step 2 | Emotional Self-Control |  | -.002 | .02 | -.01 | .05* | .05* |  |
|  |  |  | Recent Immigrant from Asia |  | -.05* | .01 | -.19 |  |  |  |
|  |  |  | Asian American |  | .02 | .02 | .07 |  |  |  |
|  |  | Step 1 | Family Recognition through Achievement |  | .01 | .02 | .01 | .0002 |  |  |
|  |  | Step 2 | Family Recognition through Achievement |  | .003 | .02 | .01 | .05* | .05* |  |
|  |  |  | Recent Immigrant from Asia |  | -.05* | .01 | -.20 |  |  |  |
|  |  |  | Asian American |  | .02 | .02 | .07 |  |  |  |
|  |  | Step 1 | Loss of Face |  | .03 | .02 | .07 | .01 |  |  |
|  |  | Step 2 | Loss of Face |  | .02 | .02 | .06 | .06* | .05* |  |
|  |  |  | Recent Immigrant from Asia |  | -.05* | .01 | -.20 |  |  |  |
|  |  |  | Asian American |  | .02 | .02 | .06 |  |  |  |
|  |  |  |  |  |  |  |  |  |  |  |

Group predictors were dummy coded so that European Americans were the reference group.

* *p* < .05
